# A novel value-based multi-criteria decision making approach to evaluate new technology adoption in SMEs

**DOI:** 10.7717/peerj-cs.1184

**Published:** 2022-12-09

**Authors:** Chetna Gupta, Jose Maria Fernandez-Crehuet, Varun Gupta

**Affiliations:** 1 Departamento de Ingeniería de Organización, Administración de Empresas y Estadística, Universidad Politécnica de Madrid, Madrid, Spain; 2 Department of Computer Science & Engineering and Information Technology, Jaypee Institute of Information Technology, Noida, India; 3 Business School, GISMA University of Applied Sciences, Potsdam, Germany; 4 Multidisciplinary Research Centre for Innovations in SMEs (MrciS), GISMA University of Applied Sciences, Potsdam, Germany

**Keywords:** Technology adoption, SMEs, Multi criteria decision making

## Abstract

The modern competition is moving quickly toward incorporating cutting-edge technological improvements to support Small and Medium Enterprises (SMEs) in enhancing their business models. Making decisions regarding implementing new technologies in SMEs is a challenging process driven by the continuous new advances in the technology industry. Important operational process decisions, such as organizational investment costs, technology acquisition, maintenance, customer experience, employee training for the proper use of each technology, reliability requirements, and security needs, must be made very carefully for such adoptions. In this research, a novel multi-criteria decision-making process model is presented to help SME decision-makers choose the optimal technology from a list of desirable choices. The proposed approach makes use of a weighted multi-criteria decision model to rank parameters by combining many criteria that are crucial in choosing the best-suited technology. A literature review and expert opinion are utilized to pinpoint key decision-makers for the adoption of technological innovation. Specialists had reviewed the proposal in the field, and early findings suggest that it might be helpful to SME decision-makers in promoting customer value and firm performance.

## Introduction

Governments have laid stress on Small and Medium Enterprises (SMEs) after the global economic crisis of 2008–2009 as important contributors to inclusive growth and long-term sustainability in the face of economic problems. Small and medium-sized businesses (SMEs) play a vital role in a country’s economy, fostering growth through increased job creation and innovation (OECD, 2019; Chatterjee & Kar, 2020). They account for over 40% growth and innovation of gross domestic product (GDP) and 70% of job creation in developing nations. Information technology (IT) has been employed to enhance the performance, reliability, and growth of small businesses due to the intricacies of their operations. IT acts as a tool to help SMEs to grow steadily, access wider markets, boost cross-national contacts, and ensure their long-term viability and transactions in the global marketplace (Dutot, Bergeron & Raymond, 2014). It facilitates a variety of social interaction methods spanning from novel business models to communication, information exchange, and cooperation for SMEs (Costa, Soares & de Sousa, 2020; Cao & Yu, 2019). Despite increased IT spending, many SMEs are ignorant of the necessity of evaluating IT investments, which has a negative impact on their technology and decision-making capacities. Nevertheless, in light of the current circumstances, it is vital to look into the best-fit technology adoption to ensure that SME performance improves. SMEs must identify and invest in innovations that will help boost their efficiency and development. Efficiency, productivity, market price, innovation, reliability, and economic edge are all areas where technology has an impact on organizational performance. The focus should be on gaining information and acquiring technology that may be used to better the situation. In recent years, research has emphasized on the need to invest proactively in digital and emerging technologies like big data analytics, cloud services, artificial intelligence, data management practices, knowledge management practices, information communication technology, blockchain technology, Internet of Things, among others by SMEs.

Despite a variety of technology adoption approaches, SMEs are still hesitant to adopt the technology (Oliveira & Martins, 2011). Furthermore, a study conducted by Mittal et al. (2018), revealed a low desire to use smart technology. Similar decision-making challenges were reported by Eze et al. (2018), which were mostly influenced by the anxiety and uncertainty of receiving the technology. This problem emerges as a result of a lack of foresight and assessment when it comes to technology adoption (Reynolds, Fourie & Erasmus, 2019). SMEs must plan and analyze technology across their business model and processes, and employ innovation as a strategy to close the technical gap and maintain competitiveness (Dyerson, Harindranath & Barnes, 2009). Many SMEs purchase technology without properly evaluating its relevance and appropriateness, resulting in behaviors that weaken businesses and place them in a precarious situation (Halicka, 2017) incorporating new technology in a business demands a thorough decision making process that involves not only personnel but also stakeholders. This is the reason why technology considerations are crucial due to the high capital expenditure and degree of uncertainty (Love et al., 2013). The decision-making strategies that set the groundwork for successful implementation and adoption play a vital role in the decision-making process responsible for the decision to deploy, incorporate, and administer new technology (Cragg, Mills & Suraweera, 2010). Because of their failure to evaluate and adopt technology, SMEs miss the opportunity to establish a competitive edge and boost their chances of survival, according to Nguyen, Newby & Macaulay (2013). According to Govender & Pretorius (2015), the motivation for technology adoption is not a cognitive perspective on an organization’s perceptions of potential savings, simplicity of use, or measure of usefulness, but rather on the strategic role technology will play in the future. Reynolds, Fourie & Erasmus (2019) suggest that using multiple evaluation models in the setting of small businesses is difficult due to SMEs’ low expertise and comprehension of business strategy. Intangible advantages, uncertainty, and other choice factors, according to Palvalin, nnqvist & Vuolle (2013), can only be assessed qualitatively. According to Serafeimidis & Smithson (2000), rather than analyzing and grasping the technology’s role, relationships, consequences, and organizational implications, more focus has been placed on prescribing how to conduct the review.

This article proposes a novel multi-criteria decision-making process model that contextualizes SMEs and is specifically designed to help technology adoption decisions that bring value to the business. It can help SME decision-makers choose the optimal technology for adoption from a list of desirable options. The proposed method uses a weighted multi-criteria decision support system to rank parameters by incorporating numerous criteria that all contribute to the best fit technology selection. It is a scalable model in which multidimensional parameters (or criteria) can be scaled according to the needs of the organization. The word “multidimensional aspect” refers to the process of determining an equivalent priority level by averaging individual weighted priorities. A literature review and expert opinion are used to identify major decision contributors to technological innovation adoption.

The rest of the article is organized as follows: The next section discusses related work followed by methodology and result discussion. Finally, the conclusion is presented at the end.

## Related work

Performance measurement creates a knowledge framework from which knowledgeable decisions can be made and defended. It is impossible for SMEs to comprehend technology’s potential without first analyzing it. Technology evaluation processes, according to Abulrub, Yin & Williams (2012) and Cowan & Daim (2011), must analyze and evaluate each technology and SME in light of their particular context or characteristics. However, a lack of such technology evaluation approaches prior to adoption and integration sometimes prevents SMEs from embracing technology that could provide them with a competitive advantage. Decision-makers must apply a thorough strategy for assessing technology in terms of industry best practices, benefits, related costs, and risks to justify investment decisions. It should also be extended to justify applicability to business processes, implementation, and organizational growth.

The rationale for technology adoption, according to Govender & Pretorius (2015) and Ghobakhloo et al. (2011), is centered on the quality commitment technology plays and an understanding of its future repercussions, rather than potential savings, simplicity of use, or a measure of usefulness in a business. The comprehensive planning and analysis of the new technology help in accessing the knowledge of the technology’s potential impact and utility to the company. However, regardless of planning, the new technology selected and the factor relationship that occurs within the dynamics of evaluating the new technology endangers the prospective benefit and realization of the benefits accruable (Halicka, 2017). Non-acceptance of technology, according to Cowan & Daim (2011) and Cragg, Mills & Suraweera (2010), is frequently due to a lack of planning and appraisal of the possibilities and restrictions associated with the adoption and utilization of new technology. Many SMEs are finding it difficult to integrate technology into their operations, although technology evaluation remains a powerful driver of technological adoption.

It is a complicated procedure that is critical to organizational processes (Serafeimidis & Smithson, 2000). As a result, rather than focusing on quantitative metrics, businesses should take a more adaptive paradigm to entrepreneurial activity that is more relevant to current business practices. The Diffusion of Innovation (DOI) and Technology–Organization–Environment (TOE) frameworks are two well-known technology adoption models that recognize the importance of decision-making and features of technology dissemination in an organizational environment (Oliveira & Martins, 2011). Economic, environmental, and social elements of robotics were analyzed and completely incorporated into the evaluation in order to establish a long-term decision-making framework for the use of robots by SME manufacturers (Epping & Zhang, 2018). Since 1989, the BEST approach, the Information Accounting Framework (INFACC), the Investment Expert System Toolkit (InVEST), IT Investment Appraisal (ITIA), and the Rigorous Appraisal and Processing of Investment Data (RAPID) have all been proposed, but none has proven to be successful. Multi-criteria decision making (MCDM) and multi-attribute decision making (MADM) are two generic acronyms for modern technology assessment methodologies that are based on computational and mathematical tools (Mardani et al., 2015). These models are based on the quantitative aspects of the technology, in which objective measurements are employed to measure the financial implications in terms of time and resources. The lack of or inadequacy of the earlier models in addressing simple and practical recommendations for SMEs in terms of technology decisions supported the idea that IT value and benefits must be assessed in individual settings in relation to observable conditions. Various models for large organizations have been built using traditional techniques, with little or no relevance to SMEs (Palvalin, nnqvist & Vuolle, 2013).

Reynolds, Fourie & Erasmus (2019) suggest that using multiple technology evaluation (TE) models in the setting of small businesses is difficult due to SMEs’ low expertise and comprehension of business strategy. Qualitative quantification is the sole way to quantify intangible benefits, uncertainty, and other decision-making considerations (Palvalin, nnqvist & Vuolle, 2013). Wiesner et al. (2018) studied four existing Small, Medium, and Micro Enterprises (SMME) maturity models for digitization and found that none of them fully provide the essential guide for adopting new digital technology. The authors propose a new model that contextualizes SMEs and is specifically designed to assist technology adoption decisions that benefit business processes.

## Methodology: material and method

### Proposed multi-criteria decision model

The evaluation process generates a knowledge basis from which informed decisions may be made and defended. It is tough for SMEs to appreciate the potential of technology without first appraising its potential. Clearly, unambiguous knowledge of technology requirements aids decision makers in selecting technology more effectively and efficiently. It can also assist decision makers in making acceptable choices between desirable options. In reality, this selection process is carried out informally by the majority of organizations. For this purpose, this article presents a novel weighted multi-criteria value based analytical method for finding the best match technology matching requirements of the organization.

### Selection of parameters

The potential parameters affecting the decision making for the adoption of technology in SMEs are carefully identified and synthesized from the literature review. A total of 10 parameters are identified and selected as the primary elements determining needs in this study. Table 1 gives a quick rundown of these variables.

**Table 1:** List of parameters and their description.

Parameter	Description
Customer experience (ease of use)	Refers to everything about a company that influences a customer’s opinion and feelings about it. It is company's connection with its customers which covers every interaction and ease of using the system. Complexity which refers to the extent to which an innovation is perceived as being challenging to understand and use also plays a critical role in its adoption and use. If integrating technology into business operations is simple, more people are likely to accept it.
Value to business and organization fit	Delivering exceptional goods and services to customers that are worth their time, effort, and money aligned with the organization’s general culture and standards. Therefore, accessing the compatibility for technology adoption must be considered with full length and breadth. Businesses must choose and deploy technology that is consistent with their internal culture and values, involving the least amount of modifications. When management of a company think technology is incompatible with the firm's current values, culture, and practices, they won’t support its adoption because it will demand a lot of learning and process adjustment. As a result, when the new technology is well-matched with the current system and culture, top management will be more supportive.
Ease of implementation and its cost	Refers to the overall ease in implementing or installing the technology and its associated total cost incurred. The biggest barrier to the adoption of technology is its complexity. When top management decides to use a technology, integration and implementation are crucial factors. Hence, the integration of a less complex technology is going help in its access and implementation.
Time and cost saving with technology	Refers to the overall benefit of derived from technology that will reduce an organization’s overall spending in terms of cost and time.
Increase in productivity	Refers to the enhancement in overall performance in terms of output from the same amount of inputs.
Cost and time for training	Refers to the cost and time it takes to train employees on new technology.
Transition and technical support	Refers to the vendor’s help during the transition and after deployment with installation, upgrades, troubleshooting, and security.
Security and reliability	Security, refers to the guarantee that the system is performing as expected, on the other hand reliability refers to the guarantee which ensures that the system is performing as expected.
Tactic advantage	An action or plan aimed at assisting someone in achieving their goals in a certain setting.
Strategic advantage	Refers to your overall ability to outcompete, as well as your returns to stakeholders including investors, employees, and communities.

**Figure 1:**
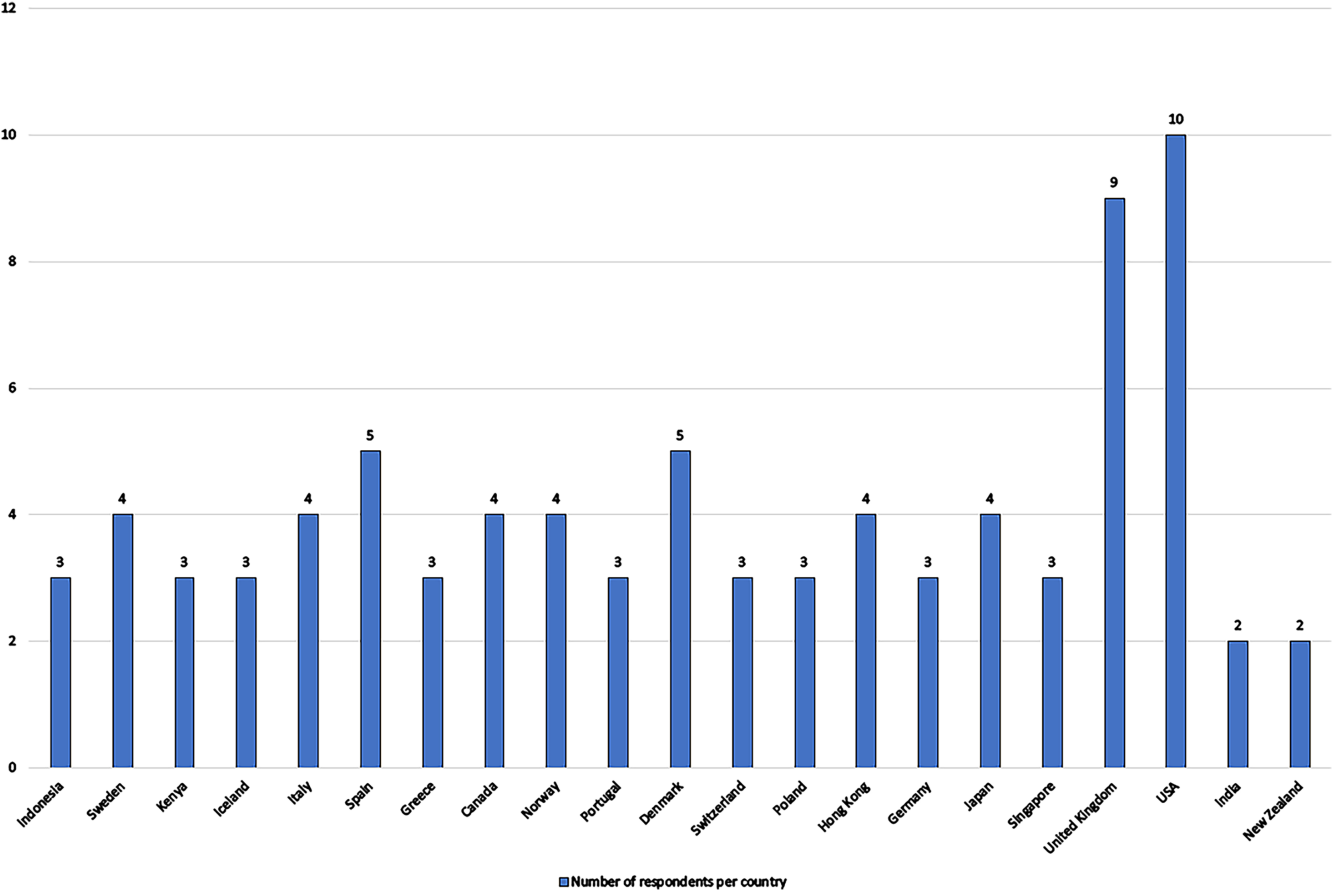
Respondents country-wise details.

Additionally, a survey was conducted with industry experts and worldwide academics with noteworthy experience for their feedback on the selection of parameters listed in Table 1. The Google forms application was used to create and publish the survey. Respondents are asked to rate the usefulness of each parameter by providing their opinion by selecting the options of “very low,” “low,” “moderate,” “high,” and “very high” for each parameter. Additionally, respondent’s work experience years, the country in which they work, and the function they play in decision making in SMEs were also recorded. The respondents work in a total of twenty one countries having a strong SME industry base. A total of 84 valid responses were collected. The profiles of the experts who took part in the survey are detailed in Table 2 and Fig. 1.

**Table 2:** Respondents experience details.

Years of experience	Respondents
Less than 3 years	8
Between 3–5 years	16
Between 5–10 years	12
Between 10–15 years	16
Between 15–20 years	13
More than 20 years	19
Number of female respondents	30%
Number of male respondents	70%

### Proposed multi-criteria value-based decision making approach

Making choices among alternatives is a vital human function that isn’t always intelligently guided and can be based on inferred or unambiguous assumptions that may or may not be accurate and absolute estimates of all the available possibilities. The following steps are followed to make a selection.

1. Decision makers carefully review potential technology alternatives for their adoption and to ensure they best fit in the organization.
2. Proposed weighted MCDM approach is applied to assess the relative value of the potential technology alternatives. The following procedure is applied:
  - Note down all of the potential technology alternatives, and attributes, that decision makers want to compare.
  - All of the items must be abstracted to the same degree. The proposed approach can handle up to a dozen of alternatives and attributes before becoming complex. In such a situation, put attributes that are related together to create a more manageable first list. Conduct a second round of analysis at a finer granularity of needs detail if necessary.
  - Assign weights to each attribute of the technology according to the relative benefit they have on the business needs. These weights can be adjusted as per requirement. Set the weighting value to 0 if no weight is to be assigned to a particular feature.
  - From the range of {Very Low, Low, Medium, High, Very High}, rate the relative advantage of each attributes to the organization or business. These benefit values show that the attributes under question are in line with the organization or business need.
  - Once all entries are complete, the final score value is assigned to each potential technology alternative using the formula:
$Attribute\left( {{a_x}} \right)\% = \displaystyle{{100*value} \over {sum\ of\ value\ attributes}}$
$Total\ Score\ ValueT\left( x \right) = \mathop \sum \nolimits_{i = 1}^n \left( {{a_x}{\rm \% }*weigh{t_x}} \right)$
  where $T\left( x \right)$ represents potential technology alternative and ${a_x}$% is relative percentage of an attributes value multiplied with its weight factor.

f. Sort the attributes list by determined score value in descending order. In contrast to other alternatives, the attributes at the top of the list have the best balance of company needs and should be adopted.

Decision maker(s) will express their preferences as linguistic values only when assigning the first priority, according to their viewpoint. Because linguistic categorizations are not as specific as numerical categorizations, linguistic values are chosen over the decision maker(s) numeric values. Decision maker(s) also find this terminology to be much closer to their expressions. Decision maker(s) may not be aware of or capable of determining the true priority value of a need. For instance, expressing “customer experience” is less specific than saying “customer experience scores first”. In such scenarios, the linguistic value of the variable describing the “priority of a criterion,” which is less specific and instructive than the numerical value “first,” is “more essential.” Despite its lack of explanation, the value "more essential" allows people to organize and present data that would otherwise be unclear or inadequate. In real-life cases, where knowledge about the technological feature is not complete and specific, which is very common, linguistic variables or values can be used as a traditional channel to portray decision maker(s) knowledge for ranking alternatives. This is the reason why the decision maker(s) will use standard linguistic terms such as {Very Low, Low, Medium, High, and Very High} to rate attributes. These terms highlight the relative importance of one criterion over another.

To better understand the meaning of these linguistic concepts, they have been mapped to an ordinal scale that can be utilized for further evaluation (refer to Table 3). The proposed model will infer information from the priority orders provided by decision maker(s) using this mapping.

**Table 3:** Respondents country-wise details.

Linguistic scale	Mapped ordinal value	Definition
Very High	1	Crucial.
High	2	Significant, but not as significant as extremely high.
Medium	3	Required, but not immediately, and have a little impact on basic functionality.
Low	4	This isn’t required right away, and it has no effect on the software’s core functionality.
Very Low	5	Basic functionality is unaffected by triviality.

**Figure 2:**
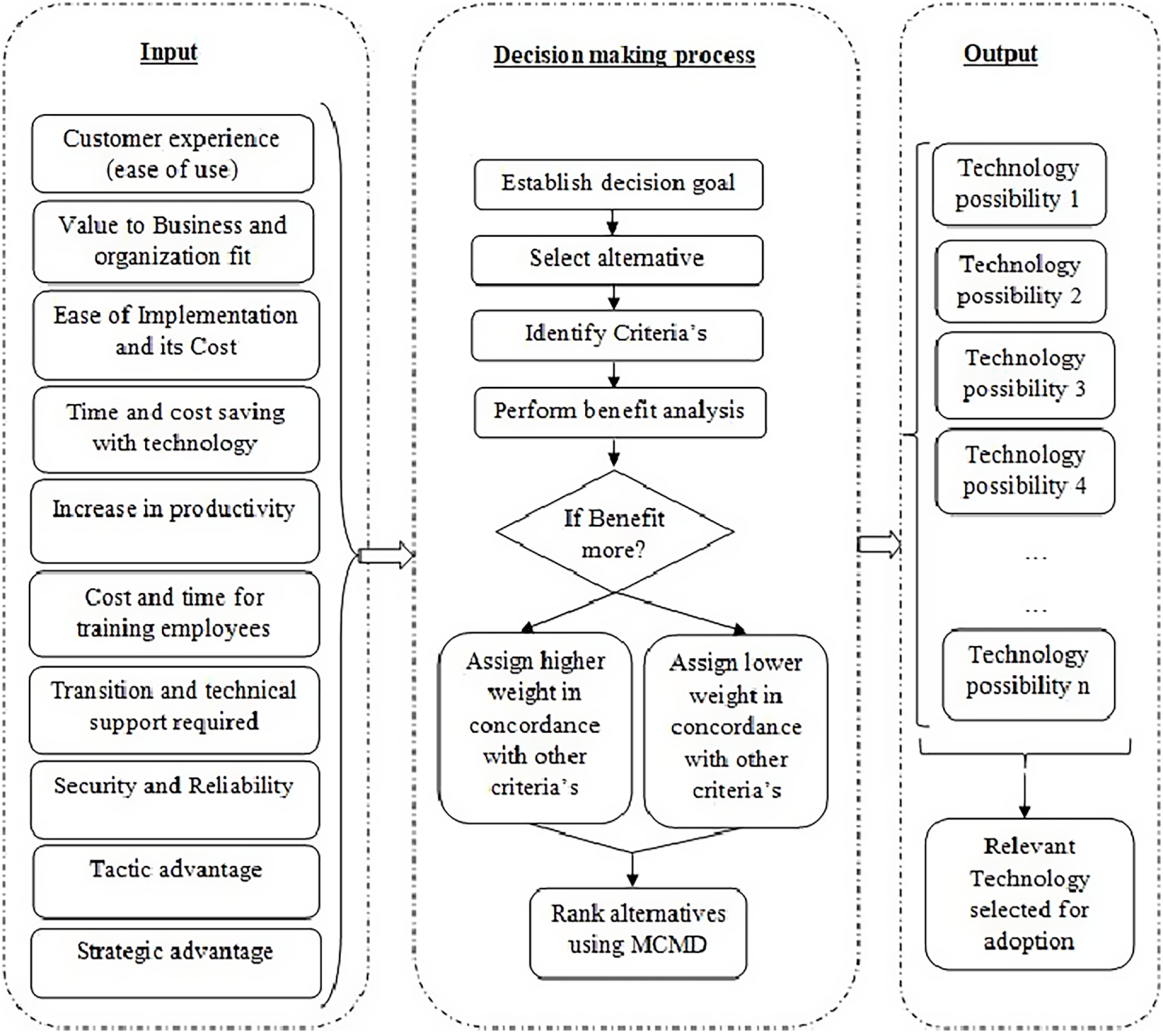
Structure of the developed weighted multi-criteria decision model.

**Figure 3:**
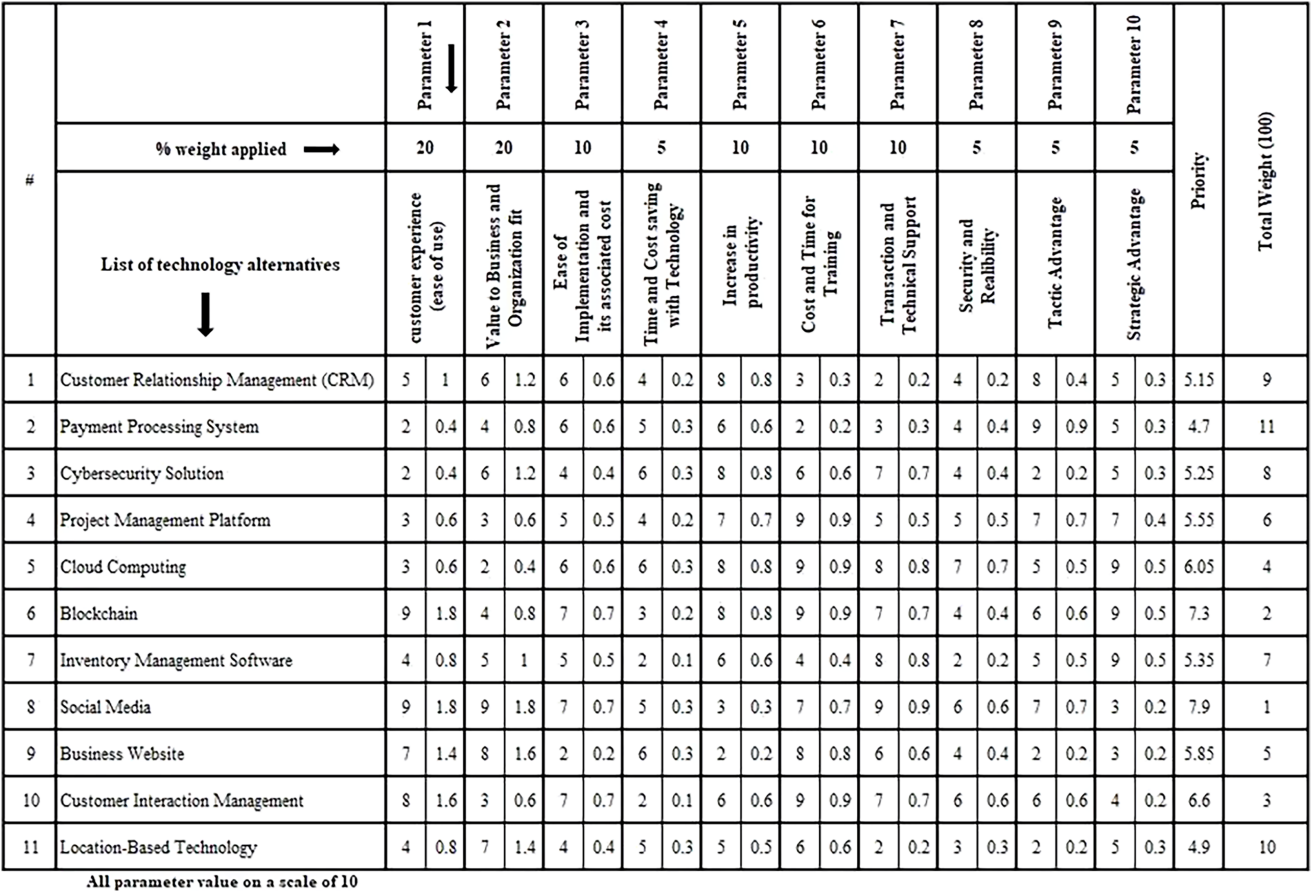
Screenshot of the template’s original version.

Figure 2 depicts the decision making process. It has ten input variables and one output variable. Figure 3 depicts a screenshot of the template’s original version. The uppermost row captures the attributes that will be used to compare the technological possibilities. A weight has been assigned to each attribute.

The value of the weight will be decided by specific organizations. Each attribute’s relative weight is calculated. Each technology choice is put in the “List of Technology alternatives” column, and each attribute is graded on the relevant scale. Referring to Fig. 3, consider a set of eleven technological alternatives which a company is considering for adoption. Each attribute of all technological alternatives is to be analyzed according to the procedure expressed by decision makers independently. The final relevance of technology is given in the last column as a priority where the values 1, 2, and 3 represent the rank of the technology in comparison to other available technology alternatives. A 1 represents the highest rank value followed by 2, 3, and so on.

### Discussion: user experience of value-based decision making approach

**Figure 4:**
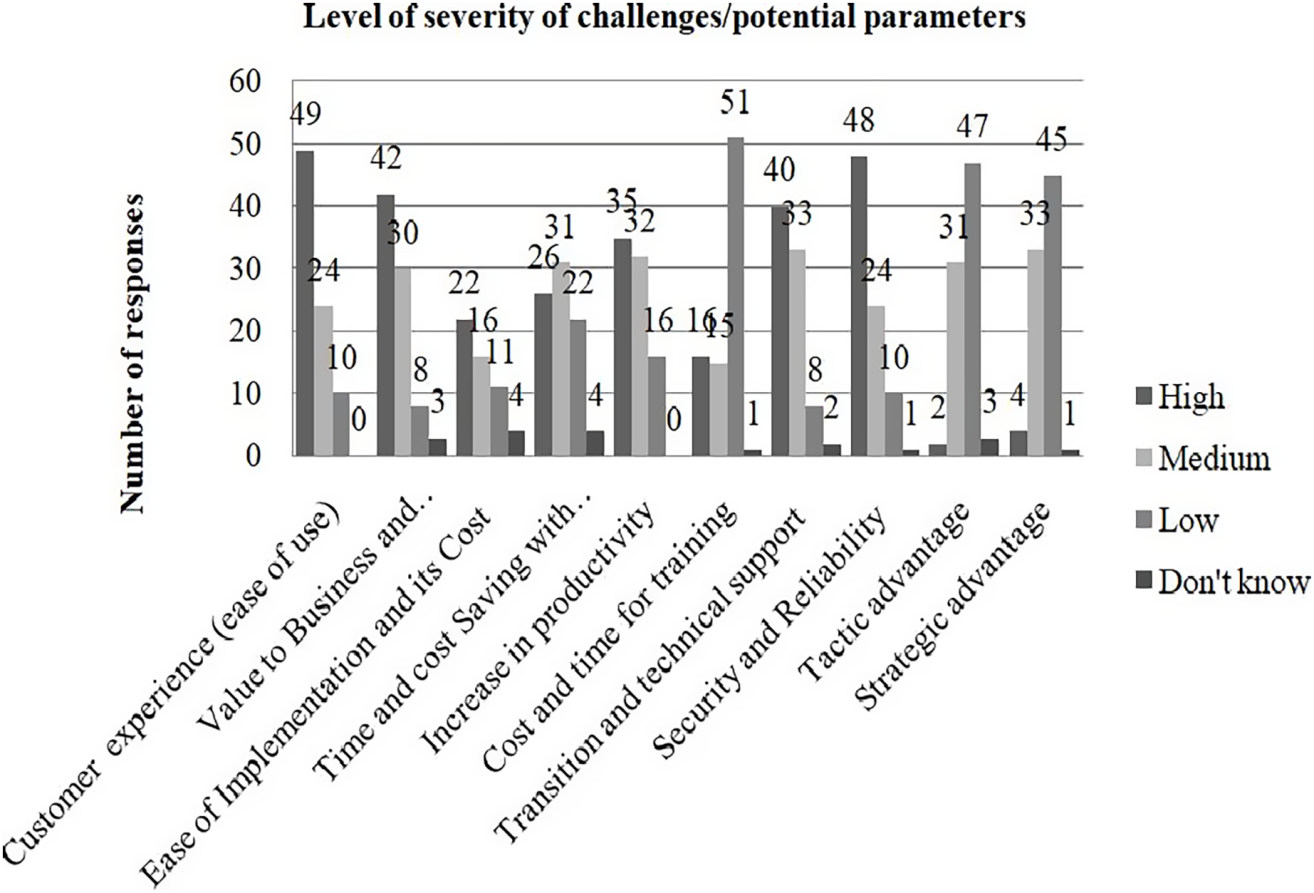
Level of severity of each challenge from responses.

In this section, a discussion on how to use a weighted multi-criteria value based decision-analysis framework is presented. The result analysis (from the data collected from decision makers, refer to Table 2) provides strong qualitative evidence of the importance of implementing the presented approach within a company. The tool was widely praised by people as being incredibly useful for making decisions at various levels of granularity within the project. Respondents who participated in the initial survey (refer to Table 2) were also asked to rate the severity of the identified potential parameters affecting the decision making for the adoption of technology in SMEs as high, moderate, and low. An additional “don’t know” response was included to reduce the noise in responses. These potential parameters are considered as challenges. Figure 4 shows the level of occurrence of each challenge from the responses.

Each participant realized the following key benefits:

- The participants agreed that the ranking of relevant technologies in many aspects was accurate and that their intuition, based on their experience, was correct. Some of the priorities they didn’t agree with (5–10 percent in most cases). The margin of error, however, was acceptable, and the proposed approach was viewed as a beneficial complement to their planning and decision-making process.
- It was discovered that using the proposed decision analysis framework reduces communication overhead when determining the relevance of technology to particular business demands. It was exceptionally quick to run a sensitivity analysis and check the resulting change in a couple of minutes, rather than having to have lengthy talks to do so. In a relatively short period, there was now a greater ability to check for numerous what-if scenarios.
- The participants were able to better focus their thoughts on the tool’s valuable elements. It was easy to determine how valuable the changes were in relation to the existing demands in the case of change in demand or needs of the organization. This supplied the organizations with solid data to use in the future about whether or not to include or remove technology and on what grounds.
- Previously, business analysts had to rely on their talents and experience to accomplish this. Using a decision analysis framework to support their decision-making process reduced the cognitive burden necessary to manage many priorities, and trade-offs, and base their decisions around high-value objectives. For these activities, the ability to handle prerequisites and have the ranking output echo a potential delivery order was extremely useful. Overall, it positively impacts organizations.

## Conclusion

In this research, we have attempted to persuade the reader that having a decision-making framework for deciding which technology is more relevant in comparison to available alternatives is truly beneficial, as it can result in significant time savings and efficient decision-making. The proposed approach helps decision makers answer the following questions:

- To assist SMEs in maximizing scope and assisting the organization in managing resources and focusing on the most valuable items first.
- In the event of fixed cost and other organizational tradeoffs, choose the greatest value technology solution to pursue.
- To assist decision makers in scoping interim decisions by focusing on and ranking the most key aspects as per organizational needs.

Although the reports acquired have positive reviews, still there are a few limitations. Most of these issues stemmed from the tool instead of the decision making framework itself. Due to the constraints of implementing them in Excel, we are working on them and attempting to alleviate them by combining them into a web-based solution. Currently, reports must be manually prepared using only raw Excel data. The participants proposed that if a reporting function allowed users to pick and select whatever types of reports they wanted, it would save even more time and assist in giving information in an easy-to-understand style. Another concern is to add a feature to better handle prerequisites (if any) while adopting technology. Despite these issues, the present version of the tool has provided useful insights into the use of decision analysis frameworks.

## Supplemental Information

10.7717/peerj-cs.1184/supp-1 Supplemental Information 1 MCMD Template.

10.7717/peerj-cs.1184/supp-2 Supplemental Information 2 Level of Severity Survey.

10.7717/peerj-cs.1184/supp-3 Supplemental Information 3 Parameter Selection Survey.

10.7717/peerj-cs.1184/supp-4 Supplemental Information 4 Level of Severity Survey Dataset.

10.7717/peerj-cs.1184/supp-5 Supplemental Information 5 Respondent Dataset.

## Additional Information and Declarations

### Data Availability

The following information was supplied regarding data availability: The live MCMD template with data from industries is available in the Supplemental Files.
